# BMI Development of Normal Weight and Overweight Children in the PIAMA Study

**DOI:** 10.1371/journal.pone.0039517

**Published:** 2012-06-28

**Authors:** Saskia M. Willers, Bert Brunekreef, Henriëtte A. Smit, Eline M. van der Beek, Ulrike Gehring, C. de Jongste, Marjan Kerkhof, Gerard H. Koppelman, Alet H. Wijga

**Affiliations:** 1 Institute for Risk Assessment Sciences (IRAS), Division Environmental Epidemiology, Utrecht University, Utrecht, The Netherlands; 2 Julius Centre for Health Sciences and Primary Care, University Medical Centre Utrecht, Utrecht, The Netherlands; 3 Danone Research, Centre for Specialised Nutrition, Wageningen, The Netherlands; 4 Danone Research, Centre for Specialised Nutrition, Singapore, Singapore; 5 Department of Pediatrics, Division of Respiratory Medicine, Erasmus University Medical Center/Sophia Children’s Hospital, Rotterdam, The Netherlands; 6 Department of Epidemiology, GRIAC Research Institute, University Medical Center Groningen, University of Groningen, Groningen, The Netherlands; 7 Department of Pediatric Pulmonology and Pediatric Allergology, GRIAC Research Institute, University Medical Center Groningen, Groningen, The Netherlands; 8 Centre for Prevention and Health Services Research, National Institute for Public Health and the Environment (RIVM), Bilthoven, The Netherlands; The University of Texas M. D. Anderson Cancer Center, United States of America

## Abstract

**Background:**

There is evidence that rapid weight gain during the first year of life is associated with overweight later in life. However, results from studies exploring other critical periods for the development of overweight are inconsistent.

**Objective:**

The objective was to investigate BMI development to assess at what ages essential differences between normal weight and overweight children occur, and to assess which age intervals the most strongly influence the risk of overweight at 8 years of age.

**Methods:**

Longitudinal weight and height data were collected by annual questionnaires in a population of 3963 children participating in the PIAMA birth cohort study. BMI and BMI standard deviation scores (SDS) were calculated for every year from birth until 8 years of age. BMI, BMI SDS and BMI SDS change in each 1-year-age interval were compared between children with and without overweight at 8 years of age, using t-tests, logistic regression analysis and the analysis of response profiles method.

**Results:**

At 8 years of age, 10.5% of the children were overweight. Already at the age of 1 year, these children had a significantly higher mean BMI SDS than normal weight 8-year-olds, (0.53 vs 0.04). In each 1-year-age interval the change in BMI SDS was significantly associated with overweight at 8 years with odds ratios increasing from 1.14 (95% CI 1.04–1.24) per 1 SDS increase at 0–1 year to 2.40 (95% CI 2.09–2.76) at 7–8 years.

**Conclusion:**

At every age, starting already in the first year of life, a rapid increase in BMI SDS was significantly associated with overweight risk at the age of 8 years. There was no evidence for a specific critical period for the development of overweight. Prevention of overweight should start early in life and be continued with age-specific interventions throughout childhood.

## Introduction

Overweight and obesity in Dutch children have markedly increased in the last decades [1]. A worrisome trend because overweight and obesity in childhood tends to track into adulthood, causing an increased risk for cardiovascular disease and type 2 diabetes [2], [3].

Prevention of overweight in childhood is high on the public health agenda, but the most promising windows of opportunity for effective intervention need to be identified [4].

Several studies have addressed the question of critical time windows for overweight development. There is now consistent evidence that growth during the first year of life is associated with overweight later in childhood, adolescence or adulthood [4]–[12]. Evidence on other periods that may be critical for overweight development is limited and unequivocal [13]–[19]. Differences in the results of these various studies may partly be related to differences in the ages at which Body Mass Index (BMI) measurements were available in the study and the length of follow-up.

Previous analyses of the PIAMA birth cohort data suggested that BMI at 1 year of age was associated with overweight at 7 years of age [20]. However, the first year of life is probably not exclusively of relevance for the development of childhood overweight.

In the present study, we studied BMI development of children with and without overweight at 8 years of age, and investigated which periods in childhood most strongly influenced the risk of overweight at 8 years of age. In contrast to earlier studies, we used regular 1-year-age intervals from birth until 8 years of age, which made it possible to study the influence of the first year of life as well as the subsequent 1-year-age intervals during childhood on the development of overweight at 8 years of age in the same way.

## Methods

### Ethics Statement

The Medical Ethical Committees of the participating institutes (Rotterdam, start project MEC 132.636/1994/39 and 137.326/1994/130; Groningen, start project MEC 94/08/92; Utrecht, start project MEC-TNO oordeel 95/50; Utrecht, age 4 years CCMO P000777C; Utrecht, age 8 years CCMO P04.0071C, protocol number 04-101/K; Rotterdam, age 8 years MEC 2004-152; Groningen, age 8 years M 4.019912; Utrecht, age 12 years METC protocol number 07-337/K) approved the study. Parents, carers or guardians gave written informed consent on behalf of all the minors/children involved in the study.

### Study Design and Population

In 1996, the large multicentre birth cohort study Prevention and Incidence of Asthma and Mite Allergy (PIAMA) was set up around three regions north (provinces Groningen, Friesland and Drenthe), central (provinces Utrecht and Gelderland) and south-west (the city of Rotterdam and some surrounding municipalities) in the Netherlands. The aim of this cohort study was to investigate the influence of different environmental and life style factors on the development of asthma and allergic disease, and to study the possibility of asthma/allergy prevention in children by the use of mite impermeable mattress covers. At baseline this cohort consisted of 4146 pregnant women, but the study started of with 3963 newborn children because 183 (4.4%) were lost to follow-up before any data of the child had been collected. Questionnaires containing questions on asthma, rhinitis and eczema as well as various questions on lifestyle factors (nutrition, pets, home characteristics etc.) were sent to the participants at 3 months of age, and annually from 1 to 8 years of age. Further details of the design of the PIAMA study have been published previously [21].

### Assessment of Weight, Height, BMI, Overweight and Obesity

Data on weight, height and date of measurement were derived from the annual questionnaires. Parents were asked to copy their child’s weight and height data and the date of measurement from their child’s medical record, or to measure their child’s weight and height, and indicate the date of measurement themselves. To facilitate longitudinal analysis of response profiles the reported weight and height data were linearly interpolated to fixed time points. This was done for measurements between boundaries of 6 months before and after the fixed time point, which were birth, 3 months, and 1, 2, 3, 4, 5, 6, 7 and 8 years of age. The interpolated weight and height data was checked for physically impossible values, which were removed. BMI (in kg/m^2^) was calculated from the interpolated weight and height data at each time point. Age and sex-specific cutoff points for BMI from the IOTF definition [22] were used to define overweight and obesity from 2 to 8 years of age. The term ‘moderate overweight’ is used to refer to the group of overweight but not obese children. BMI standard deviation scores (BMI SDS) were calculated using reference data from the 1997 Dutch Growth Study [23]. The BMI SDS thus represents the deviation in BMI from the mean BMI of the general population of children of the same age and gender.

### Missing Data and Multiple Imputation of Weight and Height Data

At 8 years of age, 8% (310/3963) of the baseline population was lost to follow up. Of the remaining children, 90% (3270) completed the 8-year questionnaire. Data on both weight and height at the same time points was available for 2958 (birth), 2287 (3 months), 2787 (1 year), 2761 (2 years), 2037 (3 years), 1944 (4 years), 1867 (5 years), 1938 (6 years), 2151 (7 years) and 2056 (8 years) children. BMI at all 10 time points was available for 489 out of the baseline population of 3963 children; 2616 children had at least 5 BMI measures. Loss-to-follow-up and missingness in the PIAMA study are unlikely to be completely at random. We used the multiple imputation method to avoid bias due to selective missing of data [24], [25]. Our longitudinal BMI dataset was imputed using the ICE (Imputation by Chained Equations) procedure implemented in Stata 11.1 (StataCorp LP., College Station, TX, USA) The predictor variables used to impute the missing weight and height data were weight and height of the child the year before and the year after the missing value, gender, gestational age, born in hospital, breastfeeding, maternal weight and height, maternal smoking during pregnancy, maternal atopy, parental educational level and region. After 100 iterations convergence was achieved resulting in 10 multiple imputed datasets, each containing the baseline population of 3963 children. These datasets were analysed using the MI analyses procedures in Stata 11.1. All analyses were done on the available case dataset and on the multiple imputed datasets. The results shown are those from the analyses in the imputed dataset.

### Statistical Analyses

The main outcome of interest was overweight or obesity at 8 years of age. Longitudinal analyses of response profiles were conducted to compare BMI and BMI SDS development from birth to 8 years of age for normal weight and overweight children at 8 years of age. The analysis of response profiles method consists of models that contain a group (overweight at 8 yes/no) and time (age) variable, and an interaction term between group and time. The model takes into account that the repeated BMI measures within one subject are correlated [26]. This analysis was used to investigate at which age BMI or BMI SDS of children with overweight at 8 years of age started to differ significantly from the BMI or BMI SDS of children without overweight at 8 years of age.

Furthermore, we compared the mean BMI SDS change between subsequent time points for normal weight versus moderately overweight and obese children at 8 years of age by means of t-tests.

This provides insight in the age intervals of BMI change that are most strongly associated with being overweight at age 8. Children with normal BMI development are expected to have a stable BMI SDS over time. A large increase in BMI SDS means that a child is gaining BMI more rapidly than expected and may become overweight or obese. Logistic regression analyses were conducted to investigate which periods most strongly influenced the risk of overweight at 8 years of age. Associations between BMI SDS at consecutive ages and overweight at 8 years of age were calculated, as well as associations between BMI SDS change between consecutive 1-year-age intervals and overweight at 8 years of age.

## Results

### Characteristics of the Study Population

Questionnaire data from the follow-up at 3 months of age and the annual follow-ups from 1 to 8 years of age were obtained for 3943, 3818, 3740, 3694, 3563, 3518, 3473, 3374 and 3270 children, respectively. Characteristics of the study population for the available case and the multiple imputed dataset (N = 3963) are shown in table 1. The prevalence of these characteristics in the imputed dataset was similar to the prevalence in the available case dataset.

**Table 1:** Characteristics of the study population for the available case and the multiple imputed dataset (N = 3963).

Available Case Dataset				Imputed Dataset	
Characteristic	N^1^	n^2^	(%)	N	(%)
Gender (male)	3963	2055	(51.9)	2055	(51.9)
Born prematurely	3930	190	(4.8)	193	(4.9)
Born in hospital	3906	2189	(56.0)	2223	(56.1)
Smoking during pregnancy	3904	696	(17.8)	707	(17.9)
Breastfeeding (≥16 weeks)	3896	1370	(35.2)	1392	(35.1)
Maternal educational level	3807				
Low		894	(23.5)	938	(23.7)
Intermediate		1582	(41.6)	1647	(41.6)
High		1331	(35.0)	1378	(34.8)
Paternal educational level	3761				
Low		973	(25.9)	1035	(26.1)
Intermediate		1295	(34.4)	1367	(34.5)
High		1493	(39.7)	1561	(39.4)
Maternal BMI >25 kg/m^2^	3171	1009	(31.8)	1313	(33.1)
Paternal BMI >25 kg/m^2^	2996	1504	(50.2)	2021	(51.0)
Atopic mother	3963	1237	(31.2)	1237	(31.2)
Atopic father	3957	1217	(30.8)	1219	(30.8)
Region	3963				
North		1231	(31.1)	1231	(31.1)
Central		1586	(40.0)	1586	(40.0)
South-west		1146	(28.9)	1146	(28.9)

1 Total available cases.

2 Number of children with specific characteristic.

### BMI Development Over Time

**Figure 1:**
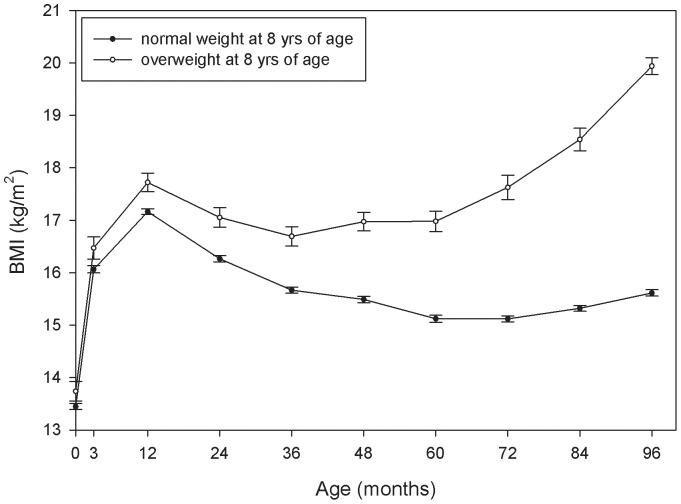
Mean (95% CI) BMI from birth to 8 years (96 months) of age for normal weight versus overweight children at 8 years of age.

**Figure 2:**
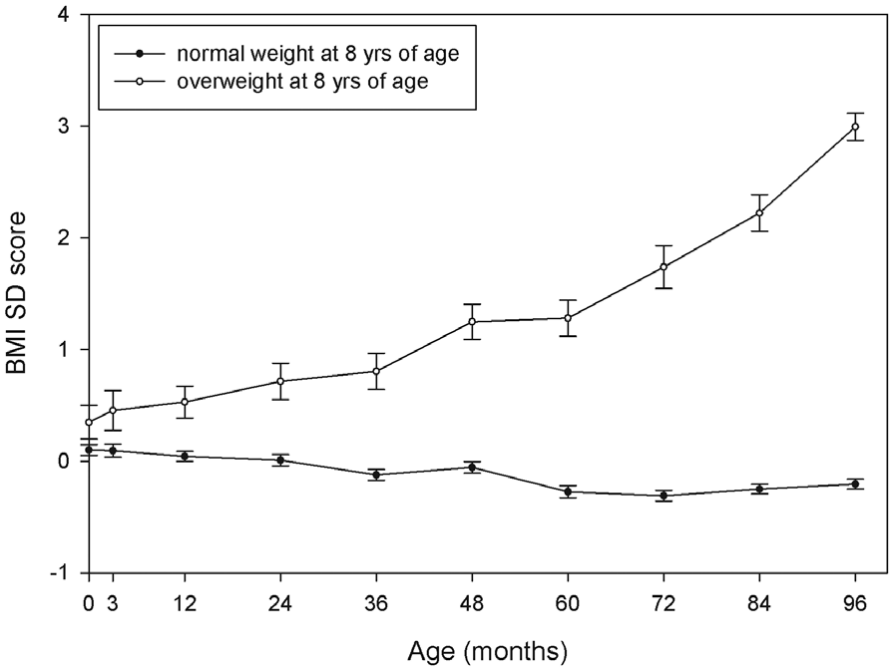
Mean (95% CI) BMI SDS from birth to 8 years (96 months) of age for normal weight versus overweight children at 8 years of age.

Table 2 shows anthropometric measures and prevalence of moderate overweight and obesity from birth to 8 years of age in the total study population. Mean weight increased from 3.51 kg at birth to 28.45 kg at 8 years of age, and mean length/height increased from 0.51 m at birth to 1.33 m at 8 years of age. BMI (kg/m^2^) increased rapidly during the first year, decreased until the age of 5 and slowly increased after the age of 5. Prevalence of moderate overweight and obesity was relatively stable at all ages and ranged from 7.2 to 9.1% and from 1.2 to 1.5% respectively. Figure 1 and 2 show the results of the analysis of response profiles method that was conducted to study the development of BMI and BMI SDS from birth to 8 years of age. BMI and BMI SDS of overweight children were higher than BMI and BMI SDS of normal weight children from birth onwards. The difference in mean BMI between children with and without overweight ranged from 0.29 kg/m^2^ at birth to 4.32 kg/m^2^ at 8 years of age, and became statistically significant from the age of 1 year onwards (figure 1). Figure 1 also shows that the mean BMI trajectory of children with overweight at 8 years of age already shows an adiposity rebound (the age at which BMI has reached its lowest point and starts to increase again) at 3 years of age, while the mean BMI trajectory of normal weight children shows a rebound that occurs between 5 and 6 years of age. The difference in mean BMI SDS between children with and without overweight ranged from 0.25 at birth to 3.20 at 8 years of age, and also became statistically significant from the age of 1 year onwards (figure 2). Figure 2 shows a steady rise of BMI SD score for the children with overweight at 8 years of age from birth to 8 years of age, that becomes even steeper from age 6 years onwards, compared to a horizontal line close to zero from birth to 8 years of age for children who were normal weight at 8 years of age.

**Table 2:** Reported weight, height and BMI development from birth to 8 years of age and prevalence of moderate overweight and obesity from 2 to 8 years of age.

Available case dataset							Multiple imputed (N = 3963)									
Age	Weight (kg)			Height (m)			Weight (kg)		Height (m)		BMI (kg/m^2^)		Moderate overweight		Obese	
	N	Mean	(SD)	N	Mean	(SD)	Mean	(SD)	Mean	(SD)	Mean	(SD)	n	(%)	n	(%)
0 (birth)	3914	3.51	(0.55)	2968	0.51	(0.03)	3.51	(0.55)	0.51	(0.03)	13.48	(1.50)	–	–	–	–
3 months	3327	6.01	(0.77)	2318	0.61	(0.03)	6.00	(0.82)	0.61	(0.03)	16.11	(1.96)	–	–	–	–
1 year	3091	10.02	(1.14)	2804	0.76	(0.03)	10.02	(1.20)	0.76	(0.03)	17.22	(1.53)	–	–	–	–
2 years	2907	12.97	(1.50)	2780	0.89	(0.04)	12.90	(1.58)	0.89	(0.04)	16.37	(1.66)	336	(8.5)	53	(1.3)
3 years	2193	15.30	(1.83)	2069	0.98	(0.04)	15.20	(1.91)	0.98	(0.05)	15.78	(1.71)	284	(7.2)	46	(1.2)
4 years	2097	17.61	(2.22)	1993	1.06	(0.04)	17.55	(2.49)	1.06	(0.05)	15.66	(1.86)	338	(8.5)	59	(1.5)
5 years	2062	19.75	(2.66)	1946	1.13	(0.05)	19.69	(3.10)	1.13	(0.06)	15.33	(2.09)	327	(8.3)	54	(1.4)
6 years	2117	22.28	(3.18)	2031	1.20	(0.05)	22.19	(3.24)	1.20	(0.06)	15.38	(1.94)	329	(8.3)	55	(1.4)
7 years	2322	25.39	(4.00)	2208	1.27	(0.05)	25.17	(4.06)	1.27	(0.06)	15.66	(2.05)	336	(8.5)	54	(1.4)
8 years	2251	28.62	(4.63)	2094	1.33	(0.06)	28.45	(5.19)	1.33	(0.07)	16.07	(2.62)	361	(9.1)	55	(1.4)

### BMI SDS Change of Normal Weight, Overweight and Obese Children at 8 Years of Age

Table 3 shows the mean BMI SDS change during consecutive 1-year-age intervals for children being normal weight, moderate overweight or obese at 8 years of age. Furthermore it shows the results of the t-tests between normal weight and moderate overweight children and normal weight and obese children, respectively, at all age intervals. Children who were moderate overweight or obese at 8 years of age had a larger increase in BMI SDS at all ages than children who were normal weight at 8 years of age. The differences in BMI SDS change between these groups were statistically significant from the age interval of 3 to 4 years onwards.

**Table 3:** Mean (SD) BMI SDS change during consecutive 1-year-age intervals for normal weight, moderate overweight and obese children at age 8 as well as results of t-test on the difference in change between these groups during these age intervals.

	BMI SDS change						T-tests for BMI SDS change in					
	Normal weight		Moderate overweight		Obese		Normal weight vs moderate overweight			Normal weight vs obese		
Age interval	Mean	(SD)	Mean	(SD)	Mean	(SD)	Coef	(SE)	p-value	Coef	(SE)	p-value
0–3 months	−0.02	(0.03)	0.09	(0.10)	0.17	(0.26)	0.11	(0.11)	0.340	0.18	(0.27)	0.517
3 months–1 year	−0.06	(0.03)	0.03	(0.10)	0.10	(0.23)	0.09	(0.10)	0.386	0.16	(0.22)	0.487
0–1 year	−0.08	(0.03)	0.12	(0.11)	0.27	(0.23)	0.20	(0.11)	0.100	0.33	(0.22)	0.137
1–2 years	−0.11	(0.02)	0.10	(0.07)	0.17	(0.22)	0.21	(0.07)	0.005	0.26	(0.18)	0.146
2–3 years	−0.03	(0.02)	0.01	(0.07)	0.16	(0.25)	0.04	(0.07)	0.651	0.18	(0.23)	0.429
3–4 years	0.07	(0.02)	0.30	(0.06)	0.62	(0.17)	0.23	(0.06)	0.001	0.53	(0.17)	0.003
4–5 years	−0.14	(0.02)	−0.02	(0.05)	0.16	(0.17)	0.12	(0.05)	0.020	0.29	(0.14)	0.041
5–6 years	−0.04	(0.02)	0.21	(0.06)	0.37	(0.21)	0.25	(0.05)	<0.001	0.39	(0.19)	0.060
6–7 years	0.06	(0.02)	0.28	(0.05)	0.39	(0.17)	0.22	(0.05)	<0.001	0.31	(0.13)	0.018
7–8 years	0.03	(0.01)	0.36	(0.05)	0.80	(0.19)	0.33	(0.04)	<0.001	0.73	(0.12)	<0.001

### Associations between BMI SDS and BMI SDS Change from Birth to 8 Years of Age and Overweight at 8 Years of Age

Table 4 shows the associations between BMI SDS (change) at different ages (1-year-age intervals) and overweight at 8 years of age. Odds ratios were calculated per 1 unit increase in BMI SDS (change). BMI SDS was significantly positively associated with overweight at 8 years of age from birth onwards. Associations between BMI SDS and overweight at 8 years became stronger with increasing age of the child. At every age interval, BMI SDS change was also significantly positively associated with overweight at 8 years of age. The odds ratio for the association between BMI SDS change between two subsequent ages and overweight at 8 years increased from 1.14 for SDS change from age 0 to 1 year, to 2.40 for SDS change from age 7 to 8 years. When including both BMI SDS and BMI SDS change as independent variables in the models (to adjust for baseline SDS at the start of the 1-year-age interval), associations became stronger and increased with increasing age of the children (data not shown). Stratification by gender showed similar results for boys and girls (table 5 and 6). The results in tables 3 and 4 and figure 1 and 2 for the multiple imputed dataset were similar to the results from the available case dataset.

**Table 4:** Associations between BMI SDS and BMI SDS change from birth to 8 years of age and overweight at 8 years of age.

BMI SDS	OR^1^	(95% CI)	p-value	BMI SDS change	OR^1^	(95% CI)	p-value
Birth	1.19	(1.06–1.34)	0.006	0–3 months	1.07	(0.98–1.16)	0.162
3 months	1.25	(1.13–1.39)	<0.001	3 months–1 year	1.07	(0.97–1.18)	0.157
1 year	1.43	(1.29–1.59)	<0.001	0–1 year	1.14	(1.04–1.24)	0.006
2 years	1.54	(1.41–1.68)	<0.001	1–2 years	1.16	(1.05–1.29)	0.005
3 years	1.71	(1.57–1.87)	<0.001	2–3 years	1.13	(1.03–1.25)	0.012
4 years	2.24	(2.02–2.49)	<0.001	3–4 years	1.32	(1.18–1.47)	<0.001
5 years	2.71	(2.41–3.05)	<0.001	4–5 years	1.26	(1.12–1.42)	<0.001
6 years	3.58	(3.15–4.07)	<0.001	5–6 years	1.50	(1.32–1.70)	<0.001
7 years	5.44	(4.57–6.48)	<0.001	6–7 years	1.56	(1.36–1.79)	<0.001
				7–8 years	2.40	(2.09–2.76)	<0.001

1 OR’s per 1 unit change in BMI SDS (change).

**Table 5:** Associations between BMI SDS and BMI SDS change from birth to 8 years of age and overweight at 8 years of age for boys (n = 2055).

BMI SDS	OR^1^	(95% CI)	p-value	BMI SDS change	OR^1^	(95% CI)	p-value
Birth	1.17	(0.99–1.39)	0.072	0–3 months	1.09	(0.98–1.21)	0.104
3 months	1.26	(1.12–1.43)	<0.001	3 months–1 year	0.98	(0.86–1.12)	0.780
1 year	1.35	(1.15–1.58)	<0.001	0–1 year	1.08	(0.95–1.24)	0.248
2 years	1.48	(1.31–1.68)	<0.001	1–2 years	1.15	(0.97–1.36)	0.103
3 years	1.77	(1.54–2.04)	<0.001	2–3 years	1.22	(1.06–1.40)	0.006
4 years	2.19	(1.89–2.54)	<0.001	3–4 years	1.28	(1.10–1.50)	0.002
5 years	2.58	(2.19–3.05)	<0.001	4–5 years	1.39	(1.17–1.65)	<0.001
6 years	3.60	(2.99–4.35)	<0.001	5–6 years	1.55	(1.26–1.89)	<0.001
7 years	5.65	(4.38–7.29)	<0.001	6–7 years	1.76	(1.46–2.13)	<0.001
				7–8 years	2.61	(2.16–3.16)	<0.001

1 OR’s per 1 unit change in BMI SDS (change)

**Table 6:** Associations between BMI SDS and BMI SDS change from birth to 8 years of age and overweight at 8 years of age for girls (n = 1908).

BMI SDS	OR^1^	(95% CI)	p-value	BMI SDS change	OR^1^	(95% CI)	p-value
Birth	1.20	1.03–1.41	0.026	0–3 months	1.06	(0.91–1.23)	0.452
3 months	1.27	1.07–1.50	0.010	3 months–1 year	1.14	(1.00–1.31)	0.059
1 year	1.49	1.31–1.70	<0.001	0–1 year	1.18	(1.05–1.33)	0.006
2 years	1.58	1.40–1.79	<0.001	1–2 years	1.19	(1.02–1.39)	0.031
3 years	1.66	1.47–1.88	<0.001	2–3 years	1.07	(0.94–1.22)	0.282
4 years	2.28	1.96–2.65	<0.001	3–4 years	1.34	(1.16–1.54)	<0.001
5 years	2.86	2.42–3.39	<0.001	4–5 years	1.16	(0.99–1.36)	0.065
6 years	3.59	2.98–4.32	<0.001	5–6 years	1.46	(1.25–1.71)	<0.001
7 years	5.48	4.31–6.97	<0.001	6–7 years	1.39	(1.14–1.70)	<0.001
				7–8 years	2.24	(1.81–2.77)	<0.001

1 OR’s per 1 unit change in BMI SDS (change)

## Discussion

Our results confirm that the risk to become overweight during childhood already starts in the first year of life. Furthermore, we showed that BMI SDS change in all subsequent years is associated with overweight later in childhood and that the associations with overweight become stronger when children grow older.

Strengths of our study were the large study population and the annually reported weight and height data so we could study BMI development from birth until 8 years of age. Detailed information on different characteristics of the study population and determinants of childhood overweight made it possible to apply the multiple imputation method to deal with missing data and loss to follow-up. We compared BMI from birth to age 8 in children with and without overweight at 8 years and investigated associations between change in BMI SDS-score during subsequent 1-year-age intervals from birth until 8 years of age and overweight at 8 years of age. Since these data were collected prospectively before assessment of overweight at 8 years of age, the likelihood of differential recall bias is limited.

A limitation of our study was that the validity of weight and height data measured by the parents might be lower than weight and height data measured by a professional. However, part of the weight and height data was derived from youth health care visits. Until the age of 45 months, Dutch children are regularly invited to the municipal child health centre to monitor the child’s health, growth and development. Height and weight are measured there with standardized methods. Parents were asked to copy their child’s weight and height measurements from their child’s medical record if possible, and to indicate whether it was measured by a professional or themselves. At the age of 4 ∼58% of the anthropometry data was measured by professionals, while after the age of 4, only 8 to 19% was measured by professionals.

At 4 and 8 years of age, weight and height was measured during medical examinations of a (sub)population of the PIAMA cohort. These measured weight and height data at 4 and 8 years of age were compared with the parental reported data at the same age. Mean differences between measured and reported weight and height data were small, but parents of children with BMI in the highest quartile underreported weight by about 0.5 kg and overreported height data by about 0.5 cm [27], [28]. This could have resulted in misclassification of overweight and therefore attenuation of our results.

Another aspect of validity of weight and height data is that they were not measured at exactly the same age in each child. Linear interpolation of the weight and height data to fixed time points could have lead to measurement error, yet it provided the opportunity to multiple impute missing data and to apply the longitudinal analysis of response profiles method [26]. Linear interpolation was done for measurements between boundaries of six months before and after the fixed time point, so model uncertainty was kept small. We have used BMI to assess overweight in our study population, while the actual outcome of interest would be total fat mass or percentage of body fat. This might have lead to measurement error. Advantages of using BMI as a measure of adiposity are that it is safe, noninvasive and inexpensive to obtain. BMI can also easily be calculated from self-reported data on weight and height and is widely used in epidemiologic research. A disadvantage of using BMI to determine overweight is that it does not distinguish between fat mass and fat-free mass. In children however, BMI is strongly associated with total fat mass [29], and considered to be a valid proxy to assess childhood overweight from 2 years of age onwards [30].

Compared to other studies the prevalence of overweight and obesity in the PIAMA cohort is relatively low. The main reason for this is that the prevalence of overweight and obesity in the Netherlands is lower than in most other Western countries. In 1997, the Dutch nationwide growth study reported a prevalence of overweight (including obesity) of 10.2% for 8-year-old boys and 14.8% for girls, while the prevalence of obesity was 1.2% and 2.3% respectively [1]. Besides possible underreporting due to the use of self-reported height and weight data, the prevalence of overweight and obesity reported in the PIAMA study is well comparable to the prevalence of overweight and obesity in the general Dutch population of the same age group.

Several earlier studies have investigated the relation between increased weight gain during specific periods and the development of overweight. A certain growth period is referred to as a critical period if changes within this period increase the risk of overweight later in life [31]. Our results are in line with studies showing that growth during the first year of life is already associated with overweight later in life [4]–[12]. In addition, we observed that growth in every subsequent 1-year-age interval contributed significantly to the risk of being overweight at the age of 8 years.

Comparison of our results to the results of other studies exploring critical periods for overweight is difficult because of differences in length of follow-up, length of time intervals and age at which overweight is defined. The Barry Caerphilly Growth study in the UK showed that weight at 5 years of age, or increased weight velocity from 1 year and 9 months to 5 years were the most important predictors for BMI, waist circumference and sagittal abdominal diameter at adult age [18]. A Finnish study showed that children who were overweight at the age of 13 years, already gained weight excessively from the age of 3 years. Girls became overweight at 5 years of age while boys became overweight at 8 years of age [17]. A study conducted in France suggests that there are 2 critical periods in early childhood that determine the risk of overweight and obesity in adolescence (8 to 17 years of age), namely the period before 6 months and the period after 2 years of age [16]. A Dutch study showed that the period between 2 and 6 years of age is the earliest and most critical growth period for adult overweight [13]. Other studies suggest that the timing of adiposity rebound is the most important predictor for later overweight [32].

The results of the present study are in line with the results cited above, but show that the relation cannot be attributed to a specific period but rather are the result of accumulating periods starting form the first year of life. We investigated BMI SDS change during 8 subsequent year-to-year intervals from birth onwards, and did not find evidence for a specific critical period for the development of overweight. At every age, starting already in the first year of life, a rapid increase in BMI SDS was significantly associated with overweight risk at the age of 8 years. In a previous study in the PIAMA population we identified a number of early life predictors of childhood overweight, including parental BMI and birth weight [33]. We also studied the influence of behavioral risk factors like diet (fast food, snack and soft drink consumption), screen time (time spent watching tv, video or at the computer) and physical activity in the PIAMA population, and found that screen time was significantly associated with overweight at 8 years of age [34]. The new observations presented in this paper suggest that in the preventive youth health care system, rapid increase in BMI SDS should also serve as a warning signal for increased overweight risk. Prevention of overweight should not exclusively be focused on the first year of life or other possible critical periods. It should start very early in life and be continued with age-specific interventions throughout childhood.
